# The Concise Guide to PHARMACOLOGY 2023/24: Introduction and Other Protein Targets

**DOI:** 10.1111/bph.16176

**Published:** 2023-10

**Authors:** Stephen P. H. Alexander, Eamonn Kelly, Alistair A. Mathie, John A. Peters, Emma L. Veale, Jane F. Armstrong, O. Peter Buneman, Elena Faccenda, Simon D. Harding, Michael Spedding, John A. Cidlowski, Doriano Fabbro, Anthony P. Davenport, Jörg Striessnig, Jamie A. Davies, Katelin E. Ahlers-Dannen, Mohammed Alqinyah, Thiruma V. Arumugam, Christopher Bodle, Josephine Buo Dagner, Bandana Chakravarti, Shreoshi P. Choudhuri, Kirk M. Druey, Rory A. Fisher, Kyle J. Gerber, John R. Hepler, Shelley B. Hooks, Havish S. Kantheti, Behirda Karaj, Somayeh Layeghi-Ghalehsoukhteh, Jae-Kyung Lee, Zili Luo, Kirill Martemyanov, Luke D. Mascarenhas, Harrison McNabb, Carolina Montañez-Miranda, Osita Ogujiofor, Hoa Phan, David L. Roman, Vincent Shaw, Benita Sjogren, Christopher Sobey, Mackenzie M. Spicer, Katherine E. Squires, Laurie Sutton, Menbere Wendimu, Thomas Wilkie, Keqiang Xie, Qian Zhang, Yalda Zolghadri

**Affiliations:** 1 School of Life Sciences, University of Nottingham Medical School, Nottingham, NG7 2UH, UK; 2 School of Physiology, Pharmacology and Neuroscience, University of Bristol, Bristol, BS8 1TD, UK; 3 School of Allied Health Sciences, University of Suffolk, Ipswich, IP4 1QJ, UK; 4 Neuroscience Division, Medical Education Institute, Ninewells Hospital and Medical School, University of Dundee, Dundee, DD1 9SY, UK; 5 Medway School of Pharmacy, The Universities of Greenwich and Kent at Medway, Anson Building, Central Avenue, Chatham Maritime, Chatham, Kent, ME4 4TB, UK; 6 Centre for Discovery Brain Sciences, University of Edinburgh, Edinburgh, EH8 9XD, UK; 7 Laboratory for Foundations of Computer Science, School of Informatics, University of Edinburgh, Edinburgh, EH8 9LE, UK; 8 Spedding Research Solutions SARL, Le Vésinet, 78110, France; 9 National Institute of Environmental Health Sciences, National Institutes of Health, Department of Health and Human Services, Research Triangle Park, NC 27709, USA; 10 PIQUR Therapeutics, Basel, 4057, Switzerland; 11 Clinical Pharmacology Unit, University of Cambridge, Cambridge, CB2 0QQ, UK; 12 Pharmacology and Toxicology, Institute of Pharmacy, University of Innsbruck, A-6020, Innsbruck, Austria; 13 University of Iowa, Iowa City, USA; 14 University of Georgia, Athens, USA; 15 National University of Singapore, Singapore, Singapore; 16 University of Pittsburgh, Iowa City, USA; 17 University of Texas Southwestern Medical Center, Dallas, USA; 18 National Institute of Health, Bethesda, USA; 19 Tetracore Inc., Athens, USA; 20 Emory University, Athens, USA; 21 University of Michigan, East Lansing, USA; 22 Cobel Darou, Shiraz, Iran; 23 Scripps Research Institute, Jupiter, USA; 24 Purdue University, West Lafayette, USA; 25 Michigan State University, East Lansing, USA; 26 La Trobe University, Clayton, Australia; 27 University of Maryland, Jupiter, USA

## Abstract

The Concise Guide to PHARMACOLOGY 2023/24 is the sixth in this series of biennial publications. The Concise Guide provides concise overviews, mostly in tabular format, of the key properties of approximately 1800 drug targets, and about 6000 interactions with about 3900 ligands. There is an emphasis on selective pharmacology (where available), plus links to the open access knowledgebase source of drug targets and their ligands (www.guidetopharmacology.org), which provides more detailed views of target and ligand properties. Although the Concise Guide constitutes almost 500 pages, the material presented is substantially reduced compared to information and links presented on the website. It provides a permanent, citable, point-in-time record that will survive database updates. The full contents of this section can be found at http://onlinelibrary.wiley.com/doi/10.1111/bph.16176. In addition to this overview, in which are identified ‘Other protein targets’ which fall outside of the subsequent categorisation, there are six areas of focus: G protein-coupled receptors, ion channels, nuclear hormone receptors, catalytic receptors, enzymes and transporters. These are presented with nomenclature guidance and summary information on the best available pharmacological tools, alongside key references and suggestions for further reading. The landscape format of the Concise Guide is designed to facilitate comparison of related targets from material contemporary to mid-2023, and supersedes data presented in the 2021/22, 2019/20, 2017/18, 2015/16 and 2013/14 Concise Guides and previous Guides to Receptors and Channels. It is produced in close conjunction with the Nomenclature and Standards Committee of the International Union of Basic and Clinical Pharmacology (NC-IUPHAR), therefore, providing official IUPHAR classification and nomenclature for human drug targets, where appropriate.

## Graphical Abstract

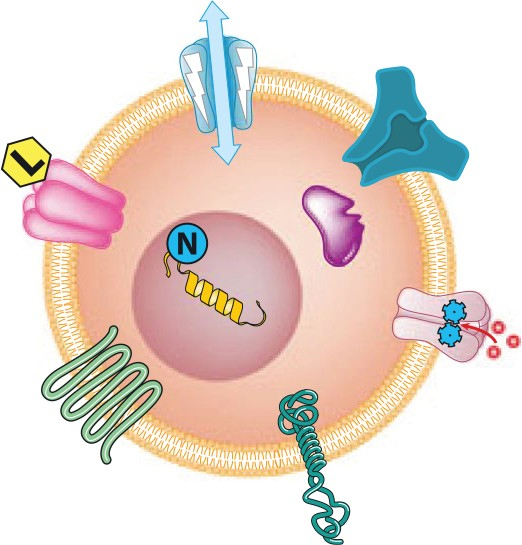

## Introduction

In order to allow clarity and consistency in pharmacology, there is a need for a comprehensive organisation and presentation of the pharmacological targets for drugs. This is the philosophy of the IUPHAR/BPS Guide to PHARMACOLOGY presented on the online free access database (https://www.guidetopharmacology.org/). This database is supported by the British Pharmacological Society (BPS), the International Union of Basic and Clinical Pharmacology (IUPHAR), the University of Edinburgh and previously the Wellcome Trust. Data included in the Guide to PHARMACOLOGY are derived in large part from interactions with the subcommittees of the Nomenclature and Standards Committee of the International Union of Basic and Clinical Pharmacology (NC-IUPHAR). A major influence on the development of the database was Tony Harmar (1951–2014), who worked with a passion to establish the curators as a team of highly informed and informative individuals, with a focus on high-quality data input, ensuring a suitably validated dataset. The Editors of the Concise Guide have compiled the individual records, in concert with the team of Curators, drawing on the expert knowledge of these latter subcommittees. The tables allow an indication of the status of the nomenclature for the group of targets listed, usually previously published in Pharmacological Reviews. In the absence of an established subcommittee, advice from several prominent, independent experts has generally been obtained to produce an authoritative consensus on nomenclature, which attempts to fit in within the general guidelines from NC-IUPHAR. This current edition, the Concise Guide to PHARMACOLOGY 2023/24, is the latest snapshot of the database in print form, following on from the Concise Guide to PHARMACOLOGY 2021/22. It contains data drawn from the online database as a rapid overview of the major pharmacological targets. Thus, there are many fewer targets presented in the Concise Guide compared to the online database. The priority for inclusion in the Concise Guide is the presence of quantitative pharmacological data for human proteins. This means that often orphan family members are not presented in the Concise Guide, although structural information is available on the online database. The organisation of the data is tabular (where appropriate) with a standardised format, where possible on a single page, intended to aid understanding of, and comparison within, a particular target group. The Concise Guide is intended as an initial resource, with links to additional reviews and resources for greater depth and information. Pharmacological and structural data focus primarily on human gene products, wherever possible, with links to HGNC gene nomenclature and UniProt IDs. In a few cases, where data from human proteins are limited, data from other species are indicated. Pharmacological tools listed are prioritised on the basis of selectivity and availability. That is, agents (agonists, antagonists, inhibitors, activators, etc.) are included where they are both available (by donation or from commercial sources, now or in the near future) AND the most selective. The Concise Guide is divided into seven sections, which comprise pharmacological targets of similar structure/function. These are G protein-coupled receptors, ion channels (including ligand-gated, voltage-gated and other ion channels), catalytic receptors, nuclear hormone receptors, enzymes, transporters and other protein targets. We hope that the Concise Guide will provide for researchers, teachers and students a state-of-the art source of accurate, curated information on the background to their work that they will use in the Introductions to their Research Papers or Reviews, or in supporting their teaching and studies. We recommend that any citations to information in the Concise Guide are presented in the following format:

Alexander SPH et al. (2023). The Concise Guide to PHARMACOLOGY 2023/24: Introduction and other protein targets. Br J Pharmacol 180: S1–S22.

In this overview are listed protein targets of pharmacological interest, which are not G protein-coupled receptors, ion channels, nuclear hormone receptors, catalytic receptors, transporters or enzymes.

## Adiponectin receptors

Other protein targets → Adiponectin receptors

### Overview:

Adiponectin receptors (**provisional nomenclature**, ENSFM00500000270960) respond to the 30 kDa complement-related protein hormone adiponectin (also known as ADIPOQ: adipocyte, C1q and collagen domain-containing protein; ACRP30, adipose most abundant gene transcript 1; apM-1; gelatin-binding protein: Q15848) originally cloned from adipocytes [74]. Although sequence data suggest 7TM domains, immunological evidence indicates that, contrary to typical 7TM topology, the carboxyl terminus is extracellular, while the amino terminus is intracellular [137]. Signalling through these receptors appears to avoid G proteins; modelling based on the crystal structures of the adiponectin receptors suggested ceramidase acivity, which would make these the first in a new family of catalytic receptors [124].

**Table:** 

Nomenclature	Adipo1 receptor	Adipo2 receptor
HGNC, UniProt	ADIPOR1, Q96A54	ADIPOR2, Q86V24
Rank order of potency	globular adiponectin (ADIPOQ, Q15848) > adiponectin (ADIPOQ, Q15848)	globular adiponectin (ADIPOQ, Q15848) = adiponectin (ADIPOQ, Q15848)

### Comments:

T-Cadherin (CDH13, P55290) has also been suggested to be a receptor for (hexameric) adiponectin [51].

## Anti-infective targets

Other protein targets → Anti-infective targets

### Overview:

This is a collection of anti-infective ligand-target interactions.

## Coronavirus (CoV) proteins

Other protein targets → Anti-infective targets → Viral protein targets → Coronavirus (CoV) proteins

### Overview:

Coronaviruses are large, often spherical, enveloped, single-stranded positive-sense RNA viruses, ranging in size from 80–220 nm. Their genomes and protein structures are highly conserved. Three coronaviruses have emerged over the last 20 years as serious human pathogens: SARS-CoV was identified as the causative agent in an outbreak in 2002–2003, Middle East respiratory syndrome (MERS) CoV emerged in 2012 and the novel coronavirus SARS-CoV-2 emerged in 2019–2020. SARS-CoV-2 is the virus responsible for the infectious disease termed COVID-19 (WHO Technical Guidance 2020).

**Table:** 

Nomenclature	CoV 3C-like (main) protease	CoV Non-structural protein 15
EC number	3.4.22.69 (SARS-CoV-2)	–
Inhibitors	nirmatrelvir (pK_i_ 9.6) [88] – SARS-CoV-2, bofutrelvir (pIC_50_ 7.3) [25] – SARS-CoV-2	tipiracil [57] – SARS-CoV-2
Comments	The Mpro enzyme (also known as nsp5 or 3CL protease) cleaves the two polyproteins encoded by the SARS-CoV-2 genome (pp1a and pp1ab) into a range of non-structural proteins (nsp1-11 from pp1a; nsp1-16 from pp1ab). As these component proteins play crucial roles in viral replication, Mpro is considered to be a strong molecular target for drug development. Small molecule Mpro inhibitors would be predicted to reduce viral replication [47, 63, 91].	Nsp15 (NendoU) is a uridylate-specific endoribonuclease that is essential during the coronavirus lifecycle. The search for inhibitors of SARS-CoV-2 nsp15 that may have antiviral action is ongoing. Two allosteric inhibitors have been reported, FUZS-5 (12200) and LIZA-7 (12199). The docking positions of these compounds within nsp15 have been determined by X-ray crystallography [34].

**Table:** 

Nomenclature	CoV Papain-like protease	CoV RNA-dependent RNA polymerase
EC number	3.4.22.46 (SARS-CoV-2)	–
Inhibitors	XR8-23 (pIC_50_ 6.4) [106] – SARS-CoV-2, GRL-0617 (pIC_50_ 5.6–5.6) [27, 86] – SARS-CoV-2	remdesivir [36] – SARS-CoV-2, remdesivir [36] – SARS-CoV
Comments	PL-pro is a domain within coronavirus Nsp3. Its proteolytic activity cleaves three sites in the viral replicase polyprotein (recognition consensus sequence LXGG↓XX) to release the three non-structural proteins Nsp1, Nsp2, and Nsp3 [44]. It has additional non-proteolytic functions as part of the multicomponent replicase-transcriptase complex [107].	The conservation of RdRP catalytic domain between different RNA viruses endows inhibitors that were designed against other viral pathogens with activity against the SARS coronaviruses. Viral RdRP is the molecular target of nucleotide-based broad-spectrum antiviral compounds like remdesivir, tenofovir and ribavirin [36, 130, 141].

**Table:** 

Nomenclature	CoV Spike glycoprotein
Inhibitors	EK-1-C4 (Binding) [136] – SARS-CoV-2
Antibodies	regdanvimab (Binding) (pK_d_ 10.6) [56] – SARS-CoV-2, casirivimab (Binding) (pIC_50_ 10.2) [42] – SARS-CoV-2
Comments	The spike protein on the surface of CoV particles is central for viral infection of host cells (by binding to ACE2). It is the molecular target of a wide range of clinically approved monoclonal antibodies that reduce infection. At any point in time, the efficacy of these therapeutics is heavily dependent upon spike mutations in the circulating CoV variants. Spike is also the antigen that’s exploited for raising anti-CoV immunity by inoculation with either mRNA and/or adenovirus vaccines that induce spike protein expression.

### Comments:

SARS-CoV-2 causes fewer fatalities than either of its predecessors MERS-CoV and SARS-CoV, but it is far more transmissible [90].

## Bacterial protein targets

Other protein targets → Anti-infective targets → Bacterial protein targets

### Overview:

Antimicrobial resistance is recognized by the World Health Organization (WHO) as a major global health threat, and it is estimated that drug-resistant infections contribute to almost 5 million deaths a year [9]. The rapid spread of bacterial strains resistant to available antibacterial medicines is of particular concern, including the ’ESKAPE’ pathogens (*Enterococcus faecium*, *Staphylococcus aureus*, *Klebsiella pneumoniae*, *Acinetobacter baumannii*, *Pseudomonas aeruginosa*, and *Enterobacter* spp.) that are responsible for many nosocomial infections [95, 123].

Antibacterial compounds act on essential bacterial molecular pathways, resulting in inhibition of growth or death of the microorganisms. These mechanisms of action include: altered DNA replication and structure, cell membrane integrity, and inhibition of cell wall peptidoglycan synthesis, nucleic acid precursor synthesis and protein synthesis.

### Complexes

**Table:** 

Nomenclature	DNA gyrase
Subunits	DNA gyrase subunit A, DNA gyrase subunit B
Comments	DNA gyrase is a type II DNA topoisomerase [31] and one of two enzymes of this subclass found in bacteria, the other being DNA topoisomerase 4. DNA gyrase introduces negative supercoils in closed circular double-stranded DNA in an ATP-dependent manner. This enzyme is the clinically-validated target for a number of antibacterial drug classes, including the aminocoumarins such as novobiocin and fluoroquinolones such as moxifloxacin, levofloxacin, ciprofloxacin and ofloxacin.

### Subunits

**Table:** 

Nomenclature	DNA gyrase subunit A	DNA gyrase subunit B
Inhibitors	ofloxacin (pIC_50_ 5.5) [12] – Escherichia coli	novobiocin (Competitive) (pIC_50_ 7.1) [6] – Escherichia coli
Comments	DNA gyrase subunit A is comprised of an N-terminal domain (59–64 kDa) involved in DNA cleavage and ligation, and a C-terminal domain (33 kDa) involved in DNA-protein interactions [93].	DNA gyrase subunit B is comprised of an N-terminal domain (43 kDa) containing the ATPase activity, and a C-terminal domain (47 kDa) involved in interactions with subunit A and DNA.

## Aryl hydrocarbon receptor

Other protein targets → Aryl hydrocarbon receptor

### Overview:

The aryl hydrocarbon receptor, highly expressed in the liver and barrier organs, is resident in the cytoplasm bound to the chaperone heat shock protein hsp90. Upon agonist activation, the ligand:aryl hydrocarbon receptor complex migrates to the nucleus and binds the aryl hydrocarbon receptor nuclear translocator (ARNT, P27540, also known as HIF1*β*). The complex regulates transcription of selected genes through interaction with xenobiotic response elements (XRE). Among the genes regulated by the AHR/ARNT complex are cytochrome P450s, particularly CYP1A1, and the period circadian protein homolog 1 (PER1, O15534). The aryl hydrocarbon receptor is also capable of non-genomic signalling.

**Table:** 

Nomenclature	Aryl hydrocarbon receptor
HGNC, UniProt	AHR, P35869
Agonists	indolo[3,2-b]carbazole [15] – Mouse, tapinarof [114], indole-3-carbinol [15] – Mouse, TCDD
Antagonists	ezutromid (pK_d_ 7.3) [134]

## Non-enzymatic BRD containing proteins

Other protein targets → Bromodomain-containing proteins → Non-enzymatic BRD containing proteins

### Overview:

Bromodomains bind proteins with acetylated lysine residues, such as histones, to regulate gene transcription. Listed herein are examples of bromodomain-containing proteins for which sufficient pharmacology exists.

**Table:** 

Nomenclature	bromodomain adjacent to zinc finger domain 2A	bromodomain adjacent to zinc finger domain 2B	CREB binding protein	polybromo 1	SWI/SNF related, matrix associated, actin dependent regulator of chromatin, subfamily a, member 4
Common abbreviation	–	–	–	–	SMARCA4
HGNC, UniProt	BAZ2A, Q9UIF9	BAZ2B, Q9UIF8	CREBBP, Q92793	PBRM1, Q86U86	SMARCA4, P51532
Inhibitors	–	–	–	GNE-064 (pIC_50_ 7.7) [125]	GNE-064 (pIC_50_ 8) [125]
Selective inhibitors	GSK2801 (pK_d_ 6.6) [104]	GSK2801 (Binding) (pK_d_ 6.9) [104]	I-CBP112 (pK_d_ 6.8) [105]	PFI-3 (Binding) (pK_d_ 7.3) [120]	PFI-3 (Binding) (pK_d_ 7.1) [120]

## CD molecules

Other protein targets → CD molecules

### Overview:

Cluster of differentiation refers to an attempt to catalogue systematically a series of over 300 cell-surface proteins associated with immunotyping. Many members of the group have identified functions as enzymes (for example, see CD73 ecto-5’-nucleotidase) or receptors (for example, see CD41 integrin, alpha 2b subunit). Many CDs are targeted for therapeutic gain using antibodies for the treatment of proliferative disorders. A full listing of all the Clusters of Differentiation proteins is not possible in the Guide to PHARMACOLOGY; listed herein are selected members of the family targeted for therapeutic gain.

**Table:** 

Nomenclature	CD2	CD3e	CD6	CD20 (membrane-spanning 4-domains, subfamily A, member 1)
HGNC, UniProt	CD2, P06729	CD3E, P07766	CD6, P30203	MS4A1, P11836
Antibodies	–	catumaxomab (Binding) [69], muromonab-CD3 (Binding) [35], otelixizumab (Binding) [17]	–	ofatumumab (Binding) (pK_d_ 9.9) [70], rituximab (Binding) (pK_d_ 8.5) [117], ibritumomab tiuxetan (Binding), obinutuzumab (Binding) [3, 94], tositumomab (Binding)

**Table:** 

Nomenclature	CD33	CD52	CD80	CD86	cytotoxic T-lymphocyte-associated protein 4 (CD152)	programmed cell death 1 (CD279)	CD300a
Common abbreviation	SIGLEC3	–	–	–	CTLA-4	PD-1	–
HGNC, UniProt	CD33, P20138	CD52, P31358	CD80, P33681	CD86, P42081	CTLA4, P16410	PDCD1, Q15116	CD300A, Q9UGN4
Endogenous ligands	–	–	–	–	–	programmed cell death 1 ligand 1 (CD274, Q9NZQ7) (Binding)	–
Antibodies	lintuzumab (Binding) (pK_d_ ~10) [19], gemtuzumab ozogamicin (Binding) [13]	alemtuzumab (Binding) [32, 108]	–	–	ipilimumab (Binding) (pK_d_ >9) [40], tremelimumab (Binding) (pK_d_ 8.9) [43]	pembrolizumab (Binding) (pK_d_ ~10) [20], nivolumab (Binding) (pK_d_ 9.1) [41, 60, 62]	–

### Comments:

The endogenous ligands for human PD-1 are programmed cell death 1 ligand 1 (CD274, Q9NZQ7) (PD-L1 *aka* CD274) and programmed cell death 1 ligand 2 (PD-L2; PDCD1LG2). These ligands are cell surface peptides, normally involved in immune system regulation. Expression of PD-1 by cancer cells induces immune tolerance and evasion of immune system attack [59]. Anti-PD-1 monoclonal antibodies are used to induce immune checkpoint blockade as a therapeutic intervention in cancer, effectively re-establishing immune vigilance. Pembrolizumab was the first anti-PD-1 antibody to be approved by the US FDA.

## Methyllysine reader proteins

Other protein targets → Chromatin reader proteins → Methyllysine reader proteins

### Overview:

Methyllysine reader proteins bind to methylated proteins, such as histones, allowing regulation of gene expression.

**Table:** 

Nomenclature	L3MBTL histone methyl-lysine binding protein 3
HGNC, UniProt	L3MBTL3, Q96JM7
Selective agonists	UNC1215 [53]

## Fatty acid-binding proteins

Other protein targets → Fatty acid-binding proteins

### Overview:

Fatty acid-binding proteins are low molecular weight (100–130 aa) chaperones for long chain fatty acids, fatty acyl CoA esters, eicosanoids, retinols, retinoic acids and related metabolites and are usually regarded as being responsible for allowing the otherwise hydrophobic ligands to be mobile in aqueous media. These binding proteins may perform functions extracellularly (*e.g*. in plasma) or transport these agents; to the nucleus to interact with nuclear receptors (principally PPARs and retinoic acid receptors [103]) or for interaction with metabolic enzymes. Although sequence homology is limited, crystallographic studies suggest conserved 3D structures across the group of binding proteins.

**Table:** 

Nomenclature	fatty acid binding protein 1	fatty acid binding protein 2	fatty acid binding protein 3	fatty acid binding protein 4	fatty acid binding protein 5
HGNC, UniProt	FABP1, P07148	FABP2, P12104	FABP3, P05413	FABP4, P15090	FABP5, Q01469
Rank order of potency	stearic acid, oleic acid > palmitic acid, linoleic acid > arachidonic acid, α-linolenic acid [96]	stearic acid > palmitic acid,oleic acid > linoleic acid > arachidonic acid, α-linolenic acid [96]	stearic acid, oleic acid, palmitic acid > linoleic acid, α-linolenic acid, arachidonic acid [96]	oleic acid, palmitic acid, stearic acid, linoleic acid > α-linolenic acid, arachidonic acid [96]	–
Inhibitors	fenofibrate (pK_i_ 7.6) [21] – Rat, fenofibric acid (pK_i_ 6.5) [21] – Rat, HTS01037 (pK_i_ 5.1) [46] – Mouse	–	–	–	compound 13 (pK_i_ 8.7) [122]
Selective inhibitors	–	–	–	HM50316 (pK_i_ >9) [71]	–
Comments	A broader substrate specificity than other FABPs, binding two fatty acids per protein [126].	Crystal structure of the rat FABP2 [99].	Crystal structure of the human FABP3 [138].	–	Crystal structure of the human FABP5 [48].

**Table:** 

Nomenclature	fatty acid binding protein 6	fatty acid binding protein 7	peripheral myelin protein 2	fatty acid binding protein 9	fatty acid binding protein 12
HGNC, UniProt	FABP6, P51161	FABP7, O15540	PMP2, P02689	FABP9, Q0Z7S8	FABP12, A6NFH5
Comments	Able to transport bile acids [142].	Crystal structure of the human FABP7 [11].	In silico modelling suggests that PMP2/FABP8 can bind both fatty acids and cholesterol [75].	–	–

**Table:** 

Nomenclature	retinol binding protein 1	retinol binding protein 2	retinol binding protein 3	retinol binding protein 4	retinol binding protein 5	retinol binding protein 7
HGNC, UniProt	RBP1, P09455	RBP2, P50120	RBP3, P10745	RBP4, P02753	RBP5, P82980	RBP7, Q96R05
Rank order of potency	–	stearic acid > palmitic acid, oleic acid, linoleic acid, α-linolenic acid, arachidonic acid [97]	–	–	–	–
Inhibitors	–	–	–	A1120 (pIC_50_ 7.8) [131]	–	–

**Table:** 

Nomenclature	retinaldehyde binding protein 1	cellular retinoic acid binding protein 1	cellular retinoic acid binding protein 2
HGNC, UniProt	RLBP1, P12271	CRABP1, P29762	CRABP2, P29373
Rank order of potency	11-cis-retinal, 11-cis-retinol > 9-cis-retinal, 13-cis-retinal, 13-cis-retinol, all-trans-retinal, retinol [24]	tretinoin > alitretinoin stearic acid > palmitic acid, oleic acid, linoleic acid, α-linolenic acid, arachidonic acid [97]	–

### Comments:

Although not tested at all FABPs, BMS309403 exhibits high affinity for FABP4 (pIC50 ~8.8) compared to FABP3 or FABP5 (pIC50 <6.6) [28, 122]. HTS01037 is reported to interfere with FABP4 action [46]. Ibuprofen displays some selectivity for FABP4 (pIC_50_ 5.5) relative to FABP3 (pIC_50_ 3.5) and FABP5 (pIC_50_ 3.8) [73]. Fenofibric acid displays some selectivity for FABP5 (pIC_50_ 5.5) relative to FABP3 (pIC_50_ 4.5) and FABP4 (pIC_50_ 4.6) [73]. Multiple pseudogenes for the FABPs have been identified in the human genome.

## Notch receptors

Other protein targets → Notch receptors

### Overview:

Aberrant Notch signalling is implicated in a number of human cancers [65, 84, 112, 128], and there is intense pharmaceutical activity being directed towards achieving clinically effective Notch pathway inhibition [26, 79].

**Table:** 

Nomenclature	notch receptor 1	notch receptor 2	notch receptor 3	notch receptor 4
HGNC, UniProt	NOTCH1, P46531	NOTCH2, Q04721	NOTCH3, Q9UM47	NOTCH4, Q99466
Inhibitors	IMR-1 (Binding) (pK_d_ 5) [10]	–	–	–
Antibodies	brontictuzumab (Binding) (pK_d_ 8.4) [37]	tarextumab (Binding) (pK_d_ >10) [38]	tarextumab (Binding) (pK_d_ 9.9) [38]	–
Comments	Various types of activating and inactivating NOTCH1 mutations have been reported to be associated with human diseases, for example: aortic valve disease [30, 78], Adams-Oliver syndrome 5 [118], T-cell acute lymphoblastic leukemia (T-ALL) [132], chronic lymphocytic leukemia (CLL) [92] and head and neck squamous cell carcinoma [1, 119].	–	–	Notch receptor 4 is a potential therapeutic molecular target for triple-negative breast cancer [66, 81].

## Regulators of G protein Signaling (RGS) proteins

Other protein targets → Regulators of G protein Signaling (RGS) proteins

### Overview:

Regulator of G protein Signaling, or RGS, proteins serve an important regulatory role in signaling mediated by G protein-coupled receptors (GPCRs). They all share a common RGS domain that directly interacts with active, GTP-bound Gα subunits of heterotrimeric G proteins. RGS proteins stabilize the transition state for GTP hydrolysis on Gα and thus induce a conformational change in the Gα subunit that accelerates GTP hydrolysis, thereby effectively turning off signaling cascades mediated by GPCRs. This GTPase accelerating protein (GAP) activity is the canonical mechanism of action for RGS proteins, although many also possess additional functions and domains. RGS proteins are divided into four families, R4, R7, R12 and RZ based on sequence homology, domain structure as well as specificity towards Gα subunits. For reviews on RGS proteins and their potential as therapeutic targets, see *e.g*. [5, 49, 83, 98, 109, 110, 111, 139, 140].

## RZ family

Other protein targets → Regulators of G protein Signaling (RGS) proteins → RZ family

### Overview:

The RZ family of RGS proteins is less well characterized than the other families. It consists of, RGS17 (also known as RGSZ2), RGS19 (also known as GAIP) and RGS20 (with several splice variants including RGSZ1 and Ret-RGS). All members contain an N-terminal cysteine string motif [68] which is a site of palmitoylation and could serve functions in membrane targeting, protein stability or aid protein-protein interactions [2, 68]. However, the function in the case of RZ family RGS proteins is not yet fully understood. Members of the RZ family of RGS proteins are the only RGS proteins that have selective GAP activity for Gα_z_, a function that resulted in the name of the family [33, 76, 129, 135]. However, the members of the RZ family are able to also GAP Gα_i/o_ members with varying selectivity.

**Table:** 

Nomenclature	regulator of G-protein signaling 17	regulator of G-protein signaling 19	regulator of G-protein signaling 20
Common abbreviation	RGS17	RGS19	RGS20
HGNC, UniProt	RGS17, Q9UGC6	RGS19, P49795	RGS20, O76081

## R4 family

Other protein targets → Regulators of G protein Signaling (RGS) proteins → R4 family

### Overview:

The R4 family of RGS proteins is the largest family of RGS proteins with 10 members. Each of the R4 family members contain only small N- and C-termini apart from the RGS domain. The N-terminal amphipathic helix present in most R4 family members serves an important function in membrane association and can directly bind phospholipids. In contrast to the RGS domain, which is well conserved among members of the R4 family of RGS proteins, the N- and C-termini vary, enabling specificity of non-GAP functions. Despite the non-complex structure of these proteins, several R4 family RGS proteins have been shown to possess additional functions apart from acting as GAPs at activated Gα subunits [14, 100].

**Table:** 

Nomenclature	regulator of G-protein signaling 1	regulator of G-protein signaling 2	regulator of G-protein signaling 3	regulator of G-protein signaling 4
Common abbreviation	RGS1	RGS2	RGS3	RGS4
HGNC, UniProt	RGS1, Q08116	RGS2, P41220	RGS3, P49796	RGS4, P49798
Selective inhibitors	–	–	–	RGS4 inhibitor 11b (pIC_50_ 7.8) [127], CCG-50014 (pIC_50_ 7.5) [16, 127], CCG-203920 (pIC_50_ 7.3) [127]

**Table:** 

Nomenclature	regulator of G-protein signaling 5	regulator of G-protein signaling 8	regulator of G-protein signaling 13	regulator of G-protein signaling 16	regulator of G-protein signaling 18	regulator of G-protein signaling 21
Common abbreviation	RGS5	RGS8	RGS13	RGS16	RGS18	RGS21
HGNC, UniProt	RGS5, O15539	RGS8, P57771	RGS13, O14921	RGS16, O15492	RGS18, Q9NS28	RGS21, Q2M5E4

## R7 family

Other protein targets → Regulators of G protein Signaling (RGS) proteins → R7 family

### Overview:

The members of the R7 family of RGS proteins [7] are more complex structures than the R4 family and are closely related to the *C. elegans* homologues EGL-10 and EAT-16 that were identified in the early stage of RGS protein research [39, 61]. Apart from the RGS domain, several additional domains are present in these proteins that mediate protein-protein interactions, sub-cellular localization and protein stability. All R7 family members form obligatory dimers with Gβ5 through the G-γ like (GGL) domain and the disheveled-EGL10-Pleckstrin homology (DEP) domain [113]. The DEP and DEP helical extension domain interact with R7 binding protein (R7BP) or RGS9 anchoring protein (R9AP; in retina) that serves as a plasma membrane anchoring mechanism [45, 54].

**Table:** 

Nomenclature	regulator of G-protein signaling 6	regulator of G-protein signaling 7	regulator of G-protein signaling 9	regulator of G-protein signaling 11
Common abbreviation	RGS6	RGS7	RGS9	RGS11
HGNC, UniProt	RGS6, P49758	RGS7, P49802	RGS9, O75916	RGS11, O94810

## R12 family

Other protein targets → Regulators of G protein Signaling (RGS) proteins → R12 family

### Overview:

The R12 family consisting of RGS10, 12 and 14. RGS12 and 14 are large proteins with additional domains that can participate in protein-protein interactions and other functions. In contrast, RGS10 is a small protein consisting of the RGS domain and small N- and C-termini, similar to members of the R4 family. However, the sequence homology the RGS10 RGS domain clearly places it in the R12 family [64]. The Gα_i/o_-Loco (GoLoco) motif in RGS12 and 14 has GDI activity (for Guanine nucleotide Dissociation Inhibitor) towards Gα_i1_, Gα_i2_ and Gα_i3_ [58, 109]. Through this activity RGS12 and RGS14 can inhibit G protein signaling both by accelerating GTP hydrolysis and by preventing G protein activation. Splice variants of RGS12 and RGS14 also contain membrane targeting and protein-protein interaction domains [101, 115, 116].

**Table:** 

Nomenclature	regulator of G-protein signaling 10	regulator of G-protein signaling 12	regulator of G-protein signaling 14
Common abbreviation	RGS10	RGS12	RGS14
HGNC, UniProt	RGS10, O43665	RGS12, O14924	RGS14, O43566

## Sigma receptors

Other protein targets → Sigma receptors

### Overview:

Although termed ’receptors’, the evidence for coupling through conventional signalling pathways is lacking. Initially described as a subtype of opioid receptors, there is only a modest pharmacological overlap and no structural convergence with the G protein-coupled receptors; the crystal structure of the sigma1 receptor [102] suggests a trimeric structure of a single short transmembrane domain traversing the endoplasmic reticulum membrane, with the bulk of the protein facing the cytosol. A wide range of compounds, ranging from psychoactive agents to antihistamines, have been observed to bind to these sites.

**Table:** 

Nomenclature	sigma non-opioid intracellular receptor 1	σ2
HGNC, UniProt	SIGMAR1, Q99720	TMEM97, Q5BJF2
Agonists	–	1,3-ditolylguanidine [67] – Guinea pig
Selective agonists	PRE-084 [121], (+)-SKF 10.047	–
Antagonists	–	SM 21 (pIC_50_ 7.2) [72]
Selective antagonists	NE-100 (pIC_50_ 8.4) [85], BD-1047 (pIC_50_ 7.4) [77]	–
Labelled ligands	[^3^H]pentazocine (Agonist)	[^3^H]-di-o-tolylguanidine (Agonist)
Comments	–	The sigma2 receptor has been reported to be TMEM97 [4], a 4TM protein partner of NPC1, the Niemann-Pick C1 protein, a 13TM cholesterol-binding protein.

### Comments:

(-)-pentazocine also shows activity at opioid receptors.

## Transthyretin

Other protein targets → Transthyretin

### Overview:

Transthyretin (TTR) is a homo-tetrameric protein which transports thyroxine in the plasma and cerebrospinal fluid and retinol (vitamin A) in the plasma. Many disease causing mutations in the protein have been reported, many of which cause complex dissociation and protein mis-assembly and deposition of toxic aggregates amyloid fibril formation [89]. These amyloidogenic mutants are linked to the development of pathological amyloidoses, including familial amyloid polyneuropathy (FAP) [8, 23], familial amyloid cardiomyopathy (FAC) [52], amyloidotic vitreous opacities, carpal tunnel syndrome [80] and others. In old age, non-mutated TTR can also form pathological amyloid fibrils [133]. Pharmacological intervention to reduce or prevent TTR dissociation is being pursued as a therapeutic strategy. To date one small molecule kinetic stabilising molecule (tafamidis) has been approved for FAP, and is being evaluated in clinical trials for other TTR amyloidoses.

**Table:** 

Nomenclature	transthyretin
Common abbreviation	TTR
HGNC, UniProt	TTR, P02766
Inhibitors	tafamidis (pK_d_ 8.7) [18]

### Comments:

Excess production and accumulation of TTR causes hereditary transthyretin-mediated amyloidosis. Two novel drugs are now approved to combat this disease: inotersen (Tegsedi) [55] and patisiran (Onpattro) [50]. Both of these drugs act to reduce the amount of TTR protein (both wild type and mutant) produced in the liver, but by slightly different mechanisms. Inotersen is an antisense oligonucleotide inhibitor of TTR synthesis, whereas patisiran is a double-stranded small interfering RNA (which targets a conserved sequence in the 3’ UTR of mutant and wild-type TTR mRNA). Inotersen is administered subcutaneously, and patisiran is delivered by intravenous infusion in a lipid nanoparticle formulation.

## Tubulins

Other protein targets → Tubulins

### Overview:

Tubulins are a family of intracellular proteins most commonly associated with microtubules, part of the cytoskeleton. They are exploited for therapeutic gain in cancer chemotherapy as targets for agents derived from a variety of natural products: taxanes, colchicine and vinca alkaloids. These are thought to act primarily through *β*-tubulin, thereby interfering with the normal processes of tubulin polymer formation and disassembly.

**Table:** 

Nomenclature	tubulin alpha 1a	tubulin alpha 4a	tubulin beta class I	tubulin beta 3 class III	tubulin beta 4B class IVb	tubulin beta 8 class VIII
HGNC, UniProt	TUBA1A, Q71U36	TUBA4A, P68366	TUBB, P07437	TUBB3, Q13509	TUBB4B, P68371	TUBB8, Q3ZCM7
Inhibitors	–	–	vinblastine (pIC_50_ 9), eribulin (pIC_50_ 8.2) [82], paclitaxel (Mitotic cell cycle arrest in A431 cells) (pEC_50_ 8.1) [87], colchicine (pIC_50_ 8) [22], cabazitaxel, docetaxel, ixabepilone, vincristine	combretastatin A4 (pIC_50_ 8.2) [29]	–	–

## References

[1] Agrawal N (2011) [21798897]

[2] Ajit SK (2007) [17126529]

[3] Alduaij W (2011) [21378274]

[4] Alon A (2017) [28559337]

[5] Alqinyah M (2018) [29042285]

[6] Alt S (2011) [21693461]

[7] Anderson GR (2009) [19521673]

[8] Andrade C (1952) [12978172]

[9] Antimicrobial Resistance Collaborators. (2022) [35065702]

[10] Astudillo L (2016) [27197169]

[11] Balendiran GK (2000) [10854433]

[12] Barnard FM (2001) [11408214]

[13] Bernstein ID. (2000) [10720144]

[14] Bernstein LS (2000) [10764749]

[15] Bjeldanes LF (1991) [1658785]

[16] Blazer LL (2011) [21329361]

[17] Bolt S (1993) [8436176]

[18] Bulawa CE (2012) [22645360]

[19] Caron PC (1992) [1458463]

[20] Carven GJ (2010)

[21] Chuang S (2008) [18533710]

[22] Cifuentes M (2006) [16504507]

[23] Coelho T (1996) [8894411]

[24] Crabb JW (1998) [9541407]

[25] Dai W (2020) [32321856]

[26] Fabbro D (2020) [33115560]

[27] Freitas BT (2020) [32428392]

[28] Furuhashi M (2007) [17554340]

[29] Gangjee A (2013) [23895532]

[30] Garg V (2005) [16025100]

[31] Gellert M (1976) [186775]

[32] Ginaldi L (1998) [9593475]

[33] Glick JL (1998) [9748279]

[34] Godoy AS (2022) bioRxiv

[35] Goldstein G (1987) [3105134]

[36] Gordon CJ (2020) [32284326]

[37] Al Gurney (2014)

[38] Gurney AL (2012)

[39] Hajdu-Cronin YM (1999) [10421631]

[40] Halk EL (2001)

[41] Hall RD (2013) [23302904]

[42] Hansen J (2020) [32540901]

[43] Hanson DC (2004)

[44] Harcourt BH (2004) [15564471]

[45] Hepler JR. (2005) [16046666]

[46] Hertzel AV (2009) [19754198]

[47] Hilgenfeld R (2014) [25039866]

[48] Hohoff C (1999) [10493790]

[49] Hollinger S (2002) [12223533]

[50] Hoy SM. (2018) [30251172]

[51] Hug C (2004) [15210937]

[52] Jacobson DR (1997) [9017939]

[53] James LI (2013) [23292653]

[54] Jayaraman M (2009) [19042037]

[55] Keam SJ. (2018) [30120737]

[56] Kim C (2021) [33436577]

[57] Kim Y (2021) [33564093]

[58] Kimple RJ (2001) [11387333]

[59] Klement JD (2023) [36917954]

[60] Kline J (2010) [21154117]

[61] Koelle MR (1996) [8548815]

[62] Korman AJ (2006)

[63] La Monica G (2022) [36169610]

[64] Lee JK (2015) [26123306]

[65] Lefort K (2007) [17344417]

[66] Lehmann BD (2015) [25993190]

[67] Lever JR (2006) [16463398]

[68] Linder ME (2007) [17183362]

[69] Linke R (2010) [20190561]

[70] Liu Q (2013)

[71] Liu X (2011) [21481589]

[72] Mach RH (1999) [10096443]

[73] Machbub B (1988) [24248795]

[74] Maeda K (1996) [8619847]

[75] Majava V (2010) [20421974]

[76] Mao H (2004) [15096504]

[77] Matsumoto RR (1995) [8566098]

[78] McBride KL (2008) [18593716]

[79] Moore G (2020) [32575680]

[80] Murakami K (1999) [10403814]

[81] Nagamatsu I (2014) [24403446]

[82] Narayan S (2011) [21324687]

[83] Neubig RR (2002) [12120503]

[84] Ntziachristos P (2014) [24651013]

[85] Okuyama S (1993) [7901723]

[86] Osipiuk J (2021) [33531496]

[87] Ouyang X (2006) [16377187]

[88] Owen DR (2021) [34726479]

[89] Penchala SC (2013) [23716704]

[90] Petersen E (2020) [32628905]

[91] Pillaiyar T (2016) [26878082]

[92] Puente XS (2011) [21642962]

[93] Reece RJ (1989) [2555327]

[94] Reslan L (2014) [23537278]

[95] Rice LB. (2008) [18419525]

[96] Richieri GV (1994) [7929039]

[97] Richieri GV (2000) [10852718]

[98] Ross EM (2000) [10966476]

[99] Sacchettini JC (1989) [2671390]

[100] Saitoh O (2001) [11087736]

[101] Schiff ML (2000) [11130074]

[102] Schmidt HR (2016) [27042935]

[103] Schroeder F (2008) [17882463]

[104] SGC. thesgc.org. Accessed on 03/03/2015.

[105] SGC. thesgc.org. Accessed on 03/03/2015.

[106] Shen Z (2021) [34665619]

[107] Shin D (2020) [32726803]

[108] Shitara K (2011)

[109] Siderovski DP (2005) [15951850]

[110] Sjögren B (2011) [21907914]

[111] Sjögren B (2010) [20691960]

[112] Sjölund J (2008) [18079963]

[113] Slepak VZ. (2009) [20374716]

[114] Smith SH (2017) [28595996]

[115] Snow BE (2002) [11771424]

[116] Snow BE (1998) [9651375]

[117] Stein R (2004) [15102696]

[118] Stittrich AB (2014) [25132448]

[119] Stransky N (2011) [21798893]

[120] Structural Genomics Consortium. http://www.thesgc.org. Accessed on 10/11/2014.

[121] Su TP (1991) [1658302]

[122] Sulsky R (2007) [17502136]

[123] Tacconelli E (2018) [29276051]

[124] Tanabe H (2015) [25855295]

[125] Taylor AM (2022) [35930799]

[126] Thompson J (1997) [9054409]

[127] Turner EM (2012) [22368763]

[128] Vilimas T (2007) [17173050]

[129] Wang J (1998) [9748280]

[130] Wang Y (2020) [32633831]

[131] Wang Y (2014) [24835984]

[132] Weng AP (2004) [15472075]

[133] Westermark P (1981) [7016817]

[134] Wilkinson IVL (2020) [31755636]

[135] Wong YH (1992) [1347957]

[136] Xia S (2020) [32231345]

[137] Yamauchi T (2003) [12802337]

[138] Young AC (1994) [7922029]

[139] Zhang P (2011) [21778436]

[140] Zhong H (2001) [11356902]

[141] Zhu W (2020) [32660307]

[142] Zwicker BL (2013) [23603607]

